# Odontological Society of Pennsylvania

**Published:** 1892-04

**Authors:** 


### ODONTOLOGICAL SOCIETY OF PENNSYLVANIA.

The usual routine business having been transacted, the President, Dr. Jack, presented the essayist of the evening, Dr. C. N. Peirce, who read a paper on "Pyorrhœa Alveolaris due largely to Systemic Predispositions."

(For Dr. Peirce's paper, see page 241.)

The discussion was opened by Dr. Truman: I take issue with Professor Peirce directly as to the origin of Pyorrhœa Alveolaris. He lays great stress upon serumal tartar. I am not aware that any one has yet demonstrated absolutely that there is such a deposit from the blood or serum upon the roots. It seems to me to be largely pure theory. We are all familiar with the depositions that may be made in all parts of the organism, and it is very possible that it may occur upon the roots of the teeth. I do not antagonize this supposed fact, but simply affirm that it has not been, in my judgment, demonstrated. Dr. Ingersoll was, I believe, the first to call attention to this tartar and its possible origin, giving it the name. If serumal tartar exists, admitting that it is present upon the roots of the teeth, what is the effect? Dr. Peirce asserts that it is primary; I entertain the view that it is secondary in its action. If I understand the origin and progress of this disease, it begins first by irritation. We find, in its minor manifestation, inflammation around the necks of the teeth, the red line indicating congestion at the marginal border, with hemorrhage upon the slightest touch.

I regard this as the beginning of this pathological condition; this necessarily becomes pyorrhœa by the increased development of pathological germs, and these I regard as the primary cause of this disturbance. When the histological conditions that exist around the necks of the teeth are considered, there will, I think, be agreement with me that the evidence is clearly made out that after the irritation is produced at this part, the development or increased development of micro-organisms takes place, and the waste products produce inflammation of the pericementum. But it is not, in my judgment, that irritation is produced by the deposit named, whether this be called salivary or serumal tartar. That it is secondary must be clear to any one who has carefully examined the condition of the pockets formed in this disease, and that irritation of the pericementum precedes that produced by the calculus present.

And again, I take exception to the fact that the deposit exists in all cases. If this were so, it would seem impossible that we should have the destructive effect which we know exists. Tartar is found in large quantities elsewhere on the teeth, but beyond producing resorption of the tissues, there is none of the usual phenomena accompanying pyorrhœa alveolaris. The absence of activity in the germ element is most noticeable; indeed, I regard the presence there in injurious numbers as an impossibility.

I must also disagree with the essayist as to the age of patients. I have had many patients with pyorrhœa that were quite young, with teeth comparatively soft. It must be admitted that it is largely a disease of middle age. My own view of the matter is that pyorrhœa is mainly local, and that it may be governed by systemic conditions which aggravate it; but if we can cure or relieve the local manifestation, the systemic will take care of themselves, so far as this disease is concerned.

The question has been raised in Professor Peirce's paper whether in the treatment of pyorrhœa alveolaris we can restore the tissues separated from the cement. It is well known that in the implantation of teeth we have reformation of bone around the tooth holding it in place. In pyorrhœa, ordinarily the pockets remain, and it becomes a difficulty as to how these are to be treated in the future, after we bring about a healthy condition. Nothing but the continual use of antiseptics will obviate this, and by their use it may be possible eventually to have redevelopment of bone. In any event they must be used, or there will be a recurrence of the disease.

The possibility of infection will depend on the transfer of irritation from one tooth to another and the continued development of micro-organisms in the new locality.

I had an interesting example of reformation of bone around teeth subsequent to treatment in this disease the present week. I treated the case over a year ago, and had no expectation that this would be satisfactory, as several of the roots were exposed almost the entire length; but during the intervening time the case was thoroughly cared for antiseptically, and, when examined, I found that, with one exception, tissue had formed closely around the margin of the gum, and the teeth were perfectly firm and healthy. This seems to show that these pockets can be filled up with osseous tissue.

*Dr. Brubaker.*—The pathological process alluded to might throw some light upon the change around the tooth. If I understand Dr. Peirce's position, there is a deposition of some sort of lime in the pericemental membrane, and that this deposition, by its presence and irritation, is the immediate cause of the flow of pus. Whether the deposition upon the roots is of the same character as the salivary calculus I do not know. There is just a possibility that the deposition, as shown by some of the teeth, is an oxalate of lime instead of phosphate. The doctor assumes that the deposit of calcareous scale is a result of either abnormal conditions of the blood-vessels, or some disorder of the general nutrition.

In urinal troubles it is well known that in the case of children of three or four years of age, there is a deposition of oxalate of lime which forms, and, being retained in this locality, it excites irritation and develops pus ; and we may have here then a condition of pyorrhœa similar to that around the teeth. This deposition of oxalate of lime might be the result either of changed condition of the blood-vessels or of change in the nutrition, and this deposition is also hereditary.

The deposition of oxalate, or any salt, in fact, always takes the line of least resistance. There is the condition which has been described as an oxalate of lime diathesis, which has many similarities to uric acid diathesis. If you take that condition, it is accumulated in the body as some disorder of nutrition. In one it is a deposition around the toe; in another around the joints of the finger and thumb ; deposit in the ears or stomach ; or it may be a deposit in the throat, according to the peculiarities of this or that individual. These may be only analogous to conditions around the teeth, and may not be at all similar; notwithstanding, I think it should be looked at from that point of view as being constitutional in character, and if that particular scale deposit on the teeth is oxalate, it would go very far to prove that it was not local in its origin, but that it comes from the blood as a result of general disorder of nutrition.

*Dr. Peirce.*—I would like to say a few words, not in answer so much as to correct a wrong impression. I thought I stated that I wanted my paper to be considered as treating pyorrhœa where it came from a deposition on the root of the tooth, called serumal or sanguinary deposit.

I have three molars in my hand which I have taken out quite recently, all coated with a hard cement on the end or near the ends of the root, from a mouth where all the teeth have been more or less affected, but these were extracted before the patient came to me, and the deposit is confined to the root of the tooth. It was simply of that phase of which I wanted to speak in the paper.

I agree with Dr. Truman that where it occurs without the presence of serumal deposition it commences at the margin of the gum; but where it comes from what has been termed serumal deposit, I am satisfied from my experience the irritation will start at the end of the root without any connection whatever with the cervical border, and from that point it works upward.

*Dr. Darby.*—First of all, I desire to congratulate the essayist upon the fact that he mentioned the name of Dr. Riggs but once in connection with the disease under consideration. We have better names than "Riggs' disease" for this condition.

I never think of his method of treatment, nor look at the instruments which he designed for the purpose, that I do not shudder, for I consider his method cruel, and in most instances futile.

I think Dr. L. C. Ingersoll was the first to denominate the deposition at the apex and along the side of the root sanguinary calculus. Possibly Dr. Black adheres to a better term when he calls it serumal calculus. It is unquestionably from the blood.

The essayist has spoken of the constitutional and hereditary origin of this disease. I can remember when it was nearly always regarded as a constitutional if not an hereditary one, and I am not sure now that the tendency to such a condition is not of this character. I will not be quoted as having said that because one or both parents have suffered from pyorrhœa alveolaris, their offspring will do so, but there have been so many instances brought to my notice where persons have said to me, my father or my mother lost their teeth in the same manner, that I am sometimes led to believe in this theory.

Professor Truman's experience is in harmony with my own observation, that the disease does not often manifest itself before middle life, and yet I have seen well-defined cases of it long before. I recall this moment a young lady in whom it appeared in a very pronounced form as early as the nineteenth year, and at twenty-six or twenty-eight she had lost all of her inferior teeth except the two wisdom teeth. I saw but yesterday a very marked and sad case of this disease. The lady had been a patient of mine for more than twenty years. Her teeth were unusually good, and she had always bestowed upon them the best of care. She has been spending three or four years in Germany and has just returned to America. My surprise was great when looking into her mouth to find all of the six anterior superior teeth elongated, loose, and pus flowing from the gingival border upon the least pressure. She, too, supposed the disease an hereditary one,—for, said she, my mother lost her teeth in this way when she was about my age.

My observation has been such as to lead me to agree with the essayist in his belief that the disease does not usually begin at the gingivæ, but at some point along the root. I am quite sure that I have seen more cases in which the disease began high up on the root than otherwise. The serumal deposit is, in my opinion, one of the sequences, and not one of the causes, of the inflammation.

*Dr. Truman.*—Dr. Peirce stated that it was not confined to one tooth. I can give an illustration in my own mouth. Twenty-five years ago I had one tooth that was affected, and treated without any success with chromic acid. I eventually relieved the difficulty, but it never extended to the adjoining teeth.

*Dr. Rehfuss.*—I have nothing especial to say upon the subject. I think with Dr. Truman that the cause of the disease is not always constitutional in its origin. I can cite in my own practice a number of cases; particularly one in which the disease had progressed and involved three teeth of the lower jaw,—two central incisors and the right lateral. In this case I had the attending physician make a thorough examination of the patient as to constitutional troubles, involving an examination of the urine and the most careful observation otherwise. While I cannot say definitely in this particular instance that it was not due to systemic troubles, still it led me to believe that such was the case. I discovered that there was a serumal deposit, but only half-way down the root; there was none at the neck; the formation of pus naturally followed, and the treatment I found brought about a normal condition of the gums, that proving to me that in this one instance it was one purely of local origin.

As to constitutional causes, a number of our best writers are agreed, as Professor Peirce has stated, that in the majority of cases it is due to urinal troubles. In this line, my investigations have not been as deep, of course, as those of the essayist, but one case in particular I can cite in which the patient had urinal trouble. I can state that it was not positively proven to my mind that the pyorrhœa alveolaris was of constitutional origin, due to this genito-urinal irritability, though there was a serumal deposit on the roots.

It seems to be a vexed and unsettled question in the profession as to whether pyorrhœa alveolaris is of constitutional or local origin. As Dr. Truman stated, constitutional troubles excite or increase the disease.

*Dr. Jeffries.*—I don't care to discuss the scientific points brought up for consideration. I have a case in mind that I would like to speak of, illustrating the fact that they are not all middle-aged patients,—one under treatment where each of the anterior lower teeth were, I think, in about the worst condition I ever saw. The gums were very much congested, teeth loose, and an instrument could be passed half-way down the roots. There was a marked depression on the lingual surface. The teeth were forced, by occlusion of the under teeth, probably one-eighth of an inch out of the arch, making it prominent and very unsightly, but by treatment with aromatic sulphuric acid, after removing the calculi, the gum healed very nicely. The right superior central had to be brought into line and held there with a gold band.

*Dr. Head.*—I desire to inquire of Dr. Peirce if he has ever seen any specimens where these deposits were found upon the teeth and did not appear at the neck? In these two specimens they are extremely interesting; it is true, the larger quantity is well below the margin of the gum, and there is a distinct line from the gum down. As regards the age, fully one-fourth of the cases which I have had have been less than twenty-eight years of age.

*Dr. Peirce.*—I have a great many cases where they were evidently, in the inception, at the apical end of the root, having no connection with the neck of the tooth. Calcic pericementitis involving at that time the end of the root only, as the exudation increases, it must find some exit, so it works out towards the border. The disease, as it progresses, must find an exit for the exudation at the root, and it finds it easier by working up and out at the gingival border than in any other way.

*Dr. Head.*—If the germs are found at the neck and at the end of the teeth, is it not difficult to tell where it starts?

*Dr. Peirce.*—I have seen many specimens where I know the commencement of this disease was at the end of the root. In its progress it made its way up to the gum-line.

*Dr. McQuillan.*—I would like the gentlemen present to tell their success in treatment. I consider I have bridled it to a considerable extent, but never made a cure of it; and after working as conscientiously as I know how and getting the mouths in fairly good condition, I find they come back in six months or a year, and then there is a slight recurrence of it, but I would like to know whether those present have had many cures. It always seems to me that a case of pyorrhœa means a continuous fee for the dentist. I always try to be very careful and keep up the treatment; not with that end in view, however, but to help the patient.

*Dr. Neal.*—I have a patient forty years of age, who I know or believe is perfectly healthy, but the right central is much affected with pyorrhœa alveolaris; the lateral is loose and projecting, and the adjoining tooth is also affected on the right anterior surface, and in each case the disease is confined only to one portion of the tooth. Pus issues from the gum. I treated it with disinfectants, and particularly phenol. I think the phenol has been of great advantage, better than hydrogen peroxide; but I am not able to cure it. The patient has been coming to me for a year or two. I am at a loss to know what to do or how to proceed. He is a man very careful, and would not lose a tooth for a great deal of money. I told him I could possibly succeed in getting the gum to unite with the tooth where it has receded. Am I correct?

*Dr. Deane.*—I am very glad there are so many here to-night who can help me out on this question. I entertained the idea a few years ago that I could cure a very difficult case, but I found the patient was worse off after treatment than before. He told me his mother had lost her teeth before she was thirty. All his teeth, without exception, are affected. He does not come as regularly as I would like to see him, and when he does, pretty nearly every tooth is bathed in pus. I have succeeded in keeping them in by using peroxide of hydrogen and iodine. I wash them out as well as I can and then inject iodine with a Lewis syringe, and in that way have been able to keep the teeth in his mouth, but they are not of much value to him.

*Dr. Peirce.*—I felt when I read the paper it would be very difficult to discriminate between irritation commencing at the gingival border and that at the end or side of the root; I wanted to discriminate between the two, and wanted my paper to apply to but one only. There is a different condition, though yielding probably to similar treatment; in both, pus and bacteria are present.

Now, so far as treatment is concerned, if I give you one typical case it will cover the ground.

About three months ago a physician in the neighborhood came to me. He is about forty-three years of age, and on looking at his mouth I was, to say the least, surprised. There was not a tooth from which there was not exuding pus, and he had thirty-two. I handed him a tumbler with a little peroxide of hydrogen, and asked him to rinse out his mouth. He washed it out repeatedly until he had it quite clean. I made an appointment for him a few days later, when I could give each tooth attention. He came at the appointed hour, and I took my scalers and went regularly around the mouth, removing the tartar as well as I could, and then applied to the gums some aristol rubbed up with gaultheria. I put it down in the pockets with a little spatula I had made from a small excavator which I had flattened at the end, so I could work it to the bottom of each pocket, and then painted the gums externally around the mouth with tincture of iodine. He wished me to see his mother; that she was seventy-five or seventy-six years of age, and was affected in the same way. She came in the course of a few days. The gentleman came back by appointment in about ten days, and I then repeated the treatment,—very similar to the first,—washing out each pocket with peroxide of hydrogen and then washing the mouth, to clear it of pus, and made an application in the same way with the gaultheria and aristol. Of course, on the second visit the gums were in very much better condition, as they had healed. The application of the remedy was successful in reducing irritation. I have had five visits from him at this time, and all his teeth have improved perceptibly; and though in better condition than they were, I cannot encourage him that his mouth will ever get well, yet I informed him that if he will keep the teeth under treatment for years, I have no doubt they will be saved, but only by constant care; to let them go five or six months without attention would be destructive to them.

The mother came in by appointment. She had lost two molars of the right side, inferior and superior, and one on the left; the remaining were somewhat loose, the bicuspids were quite loose, and the anterior teeth all had little pockets. I treated her the second time; her mouth is in a much improved condition. I never saw a more rapid and marked change in the mouth of any one. The two first molars are the only ones I fear losing. The others have healed, and while the pockets are there, there is no exudation; but I told her she will have to come every two months, as that is the only way she can keep them.

There was a little tendency in the son to salivary deposit, but the free use of oranges abated it; but on the necks of the teeth there was irritation on the cementum on nearly every tooth, but whether that was due to the frequent use of oranges I could not say. It may have been only a concomitant and not the result. When he first visited me his teeth had given him considerable pain, but after the first treatment he had none.

In speaking of gaultheria and aristol, I have a preparation that I like, because I can make up a quantity of it and have it for use.

I take the aristol in a wide-mouthed bottle and put in equal quantities of tincture of iodine, oil of gaultheria, oil of cinnamon, and carbolic acid. It has an advantage over gaultheria and aristol. It is not gummy; the iodine cuts it and makes it creamy, so that it can be readily used, and it remains without change for days or weeks. I like the preparation, and have used it for some time.

Dr. Luckie asked what would be the result of a patient using Buffalo lithia water, stating that he had been asked the question by a patient, and he replied that he did not know.

*Dr. Darby.*—I do not think my method of treatment is unlike that of many others. I have been using of late, with much satisfaction, "Dr. Robinson's remedy," which consists of carbolic acid and caustic potash, and after the manner suggested by the gentleman whose name the remedy bears. It is simply to remove as much of the deposit as possible, and then pack into the pockets cotton saturated with this remedy. I have seen some wonderful changes brought about in a few days by one or two applications made as above indicated. I leave the cotton in the pockets as long as it will remain, which is usually two or three days.

*Dr. Deane.*—In speaking of instruments, I would like to suggest that the ordinary quill tooth-pick can be satisfactorily used by splitting off half an inch of it and inserting it in an ordinary nerve-broach carrier. With it you can carry medicine up the side of the tooth nicely. It goes almost up to the apex, because you can shave it down and use a pumping motion, which carries the remedy up the side of the tooth by capillary attraction; after using it can be thrown aside and another made.

*Dr. Luckie.*—I would ask what effect the destruction of the pulp would have? Dr. Peirce has spoken of the dentine filling up during the progress of the disease, and I would be pleased to know if devitalization of the pulp would have any effect in changing nutrition from the pulp, if it was diverted to the peridental membrane. I am treating a case where I intend to fasten all the teeth together. There has been considerable abrasion of the teeth, and the cusps of the bicuspids are entirely worn away; the remaining teeth it is my intention to devitalize. I would ask to have the opinion of the doctor on that method of treatment.

*Dr. Peirce.*—I remember Dr. Garretson coming to me some ten years ago and saying his method of treating it was to devitalize the tooth; he then made some further experiments, and a few months later reported that he had not had the satisfaction he had anticipated from this method. I don't know of any other one who ever tried it. That is all I know of the matter. I never attempted it myself, but don't think it would be beneficial.

*Dr. McQuillan.*—The worst case I have had in my practice is one that has been in my hands for fifteen years, and my father's for twenty-five. I have seen the patient every six months. About five years ago there were signs of pyorrhœa, and it has been very difficult to save the tooth ; the disease has progressed rapidly.

I have used Dr. Robertson's remedy as mentioned by Dr. Darby, and have found most gratifying results from it; and also where I have an intelligent patient I also give them a preparation of chloride of zinc, six or eight grains to an ounce of rose-water, and tell them to paint the gums with a camel's-hair pencil once or twice a week. I have found considerable benefit in that direction.

*Dr. Jack.*—I have little that will add to what has already been said. I would speak of one case where I tried successfully to retain the teeth, where the disease had occasioned but slight separation of the softer tissues. We may be able to preserve them for a considerable length of time, and yet where deeper pockets have occurred, in consequence of the longer-continued disease, the obstacle in the way of amelioration would appear to my mind to be due to the fact that it is impossible to produce a state of permanent cleanliness in the pockets, they being filled with the *débris* of substances in mastication, and also by exudations that cannot by any means escape.

*Dr. Rehfuss.*—My method of treatment does not differ possibly with that of other practitioners. I first remove all deposits, if there be any, on the roots of the teeth, and wash them out, using permanganate of potash or hydrogen peroxide to get rid of all pus, and then I have recourse to massage, possibly after my second treatment. I use three forms of massage; following this I use a paste of aristol or iodoform or iodol,—either one of the three in connection with oil of cassia,—and place it in the pus pockets. I find massage treatment very beneficial, but it must be very gentle at first.

I wish to say that Dr. Mills, of New York, has experimented with massage in pyorrhœa alveolaris since I first brought out my papers on the subject, and he claims he has much better success than he did before using it.

On motion of Dr. McQuillan, the thanks of the Society were extended to Dr. Peirce.

Adjourned.

